# Supplementary material for: PRC2-EZH1 contributes to circadian gene expression by orchestrating chromatin states and RNA polymerase II complex stability
Source: EMBO J. 2024 Oct 21;43(23):6052–75. doi: 10.1038/s44318-024-00267-2 (PMC11612306; doi:10.1038/s44318-024-00267-2)
Supplement: Supplementary file 1 — Appendix [file 44318_2024_267_MOESM1_ESM.pdf]

Appendix Figures for

PRC2- EZH1 contributes to circadian gene expression by orchestrating chromatin states and RNA polymerase II complex stability

Peng Liu, Seba Nadeef, Maged F. Serag, Andreu Paytuví-Gallart, Maram Abadi, Francesco Della Valle, Santiago Radio, Xenia Roda, Jaïr Dilmé Capó, Sabir Adroub, Nadine Hosny El Said, Bodor Fallatah, Mirko Celii, Gian Marco Messa, Mengge Wang, Mo Li, Paola Tognini, Lorena Aguilar-Arnal, Satoshi Habuchi, Selma Masri, Paolo Sassone-Corsi, & Valerio Orlando

* Corresponding authors E-mail: peng.liu@kaust.edu.sa, valerio.orlando@kaust.edu.sa

Table of Content:

Appendix Figure S1 ---Page 2
Appendix Figure S2 ---Page 4
Appendix Figure S3 ---Page 6
Appendix Figure S4 ---Page 7
Appendix Figure S5---Page 8
Appendix Fig. S6---Page 9
Appendix Fig. S7---Page 10
Appendix Fig. S8---Page 11
Appendix Fig. S9---Page 12
Appendix Fig. S10---Page 13
Appendix Fig. S11---Page 14
Appendix Fig. S12---Page 15
Appendix Fig. S13---Page 16
Appendix Fig. S14---Page 17
Appendix Fig. S15---Page 19
Appendix Fig. S16---Page 21

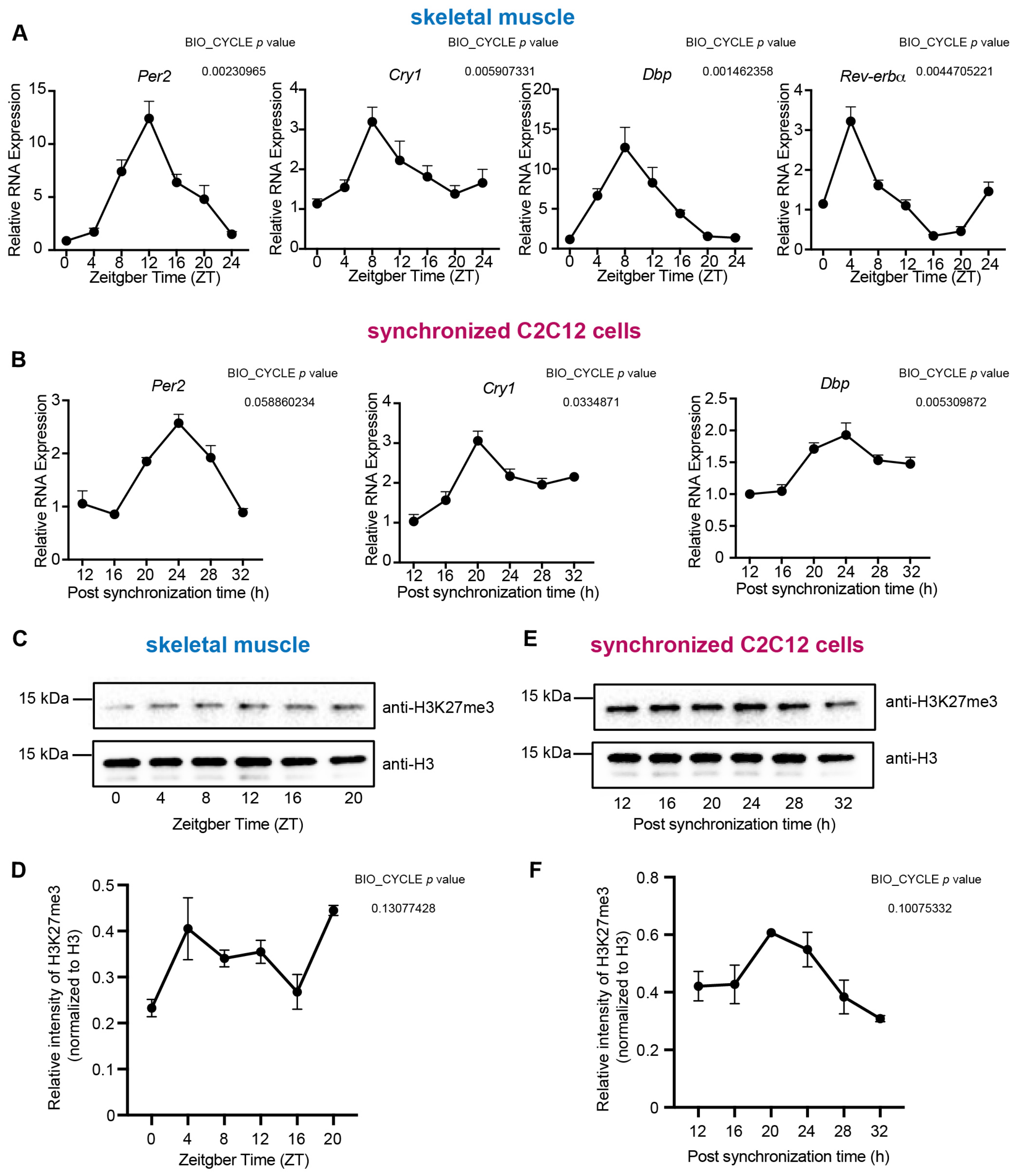

Appendix Figure S1. Expression of other core clock genes and Levels of H3K27me3 in skeletal muscle tissue and synchronized C2C12. (A) Mice were sacrificed at 4 hr intervals. Circadian profile of other clock components *Per2*, *Cry1*, *Rev-erbα , and Dbp*, RNA from mice gastrocnemius muscle tissue was analyzed by qPCR using specific oligos (n= 5 per time point). Error bars represent ± SEM from five independent biological experiments. **(B)** Circadian profile of *Per2*, *Dbp,* and *Cry1*. RNA from synchronized C2C12 was analyzed by qPCR using specific oligos (n= 3 per time point). Error bars represent ± SEM from three independent biological experiments. **(C)** Levels of H3K27me3 in mouse gastrocnemius muscle tissue were analyzed by immunoblotting. Three independent experiments were performed, and representative data was shown. Global H3 was used as an internal control. **(D)** Quantitative analysis of protein abundance of H3K27me3

shown in panel (**C)** in gastrocnemius muscle tissue. Error bars represent ± SEM from three independent biological experiments. **(E)** Levels of H3K27me3 from synchronized C2C12 cells at indicated time points were analyzed by immunoblotting. Three independent biological experiments were performed, and representative data was shown. Global H3 was used as an internal control. **(F)** Quantification of protein abundance of H3K27me3 shown in panel (**E**). Error bars represent ± SEM from three independent biological experiments. Circadian *P* values indicated from panels (**A)** to (**F**) represent rhythmic transcripts or proteins using the non-parametric test Bio-cycle, where $P < 0.05$ is considered statistically significant, incorporating a window of 24hr (muscle tissue) and 32hr (C2C12).

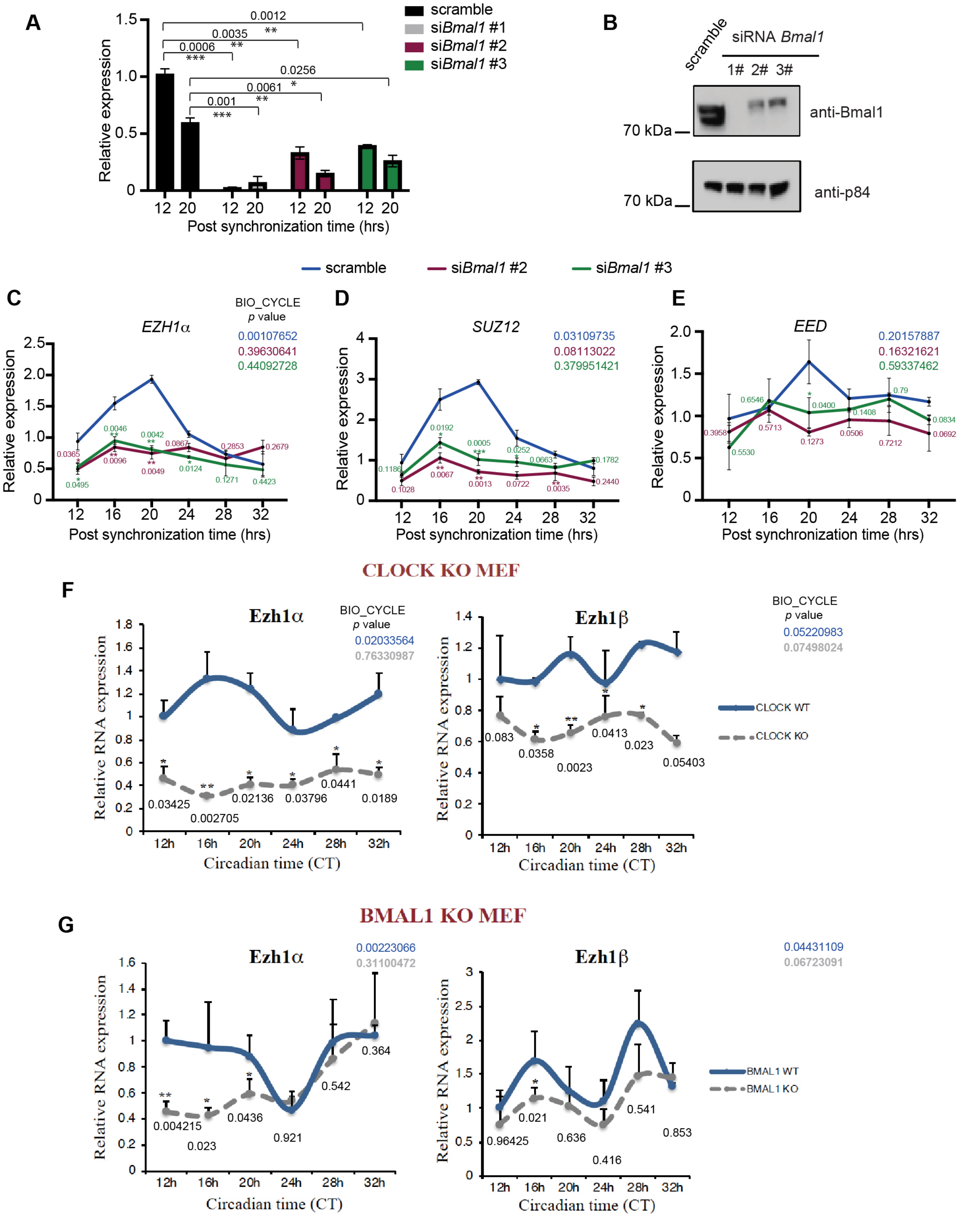

Appendix Figure S2. BMAL1/CLOCK complex is required for *Ezh1* and *Suz12* oscillation in MEF cells. (A, B) RT-qPCR and immunoblotting of knock-down efficiency of *Bmal1*/BMAL1 in C2C12 treated with different siRNAs against *Bmal1* during the circadian period. Error bars represent ± SEM from three independent biological experiments. *$p < 0.05$; **$p < 0.01$; ***$p < 0.001$; by two-tailed t test. **(C-E)** Circadian pattern of *Ezh1α, Suz12 and EED* was measured from synchronized *C2C12* cells treated with scramble or siRNAs (#2 and #3) targeting *Bmal1* at

indicated time points and analyzed by quantitative real-time PCR using specific oligos (n= 3 per time point). Error bars represent ± SEM from three independent replicates. *$p < 0.05$; **$p < 0.01$; ***$p < 0.001$; by two-tailed *t* test. (**F, G**) Circadian profile of *Ezh1* α*, Suz12 and EED* was measured from synchronized *Clock* KO (**f**) or *Bmal1* KO (**g**) MEF cells at indicated time points and analyzed by quantitative real-time PCR using specific oligos (n= 3 per time point). Error bars represent ± SEM from three independent replicates. *$p < 0.05$; **$p < 0.01$; by two-tailed *t* test.

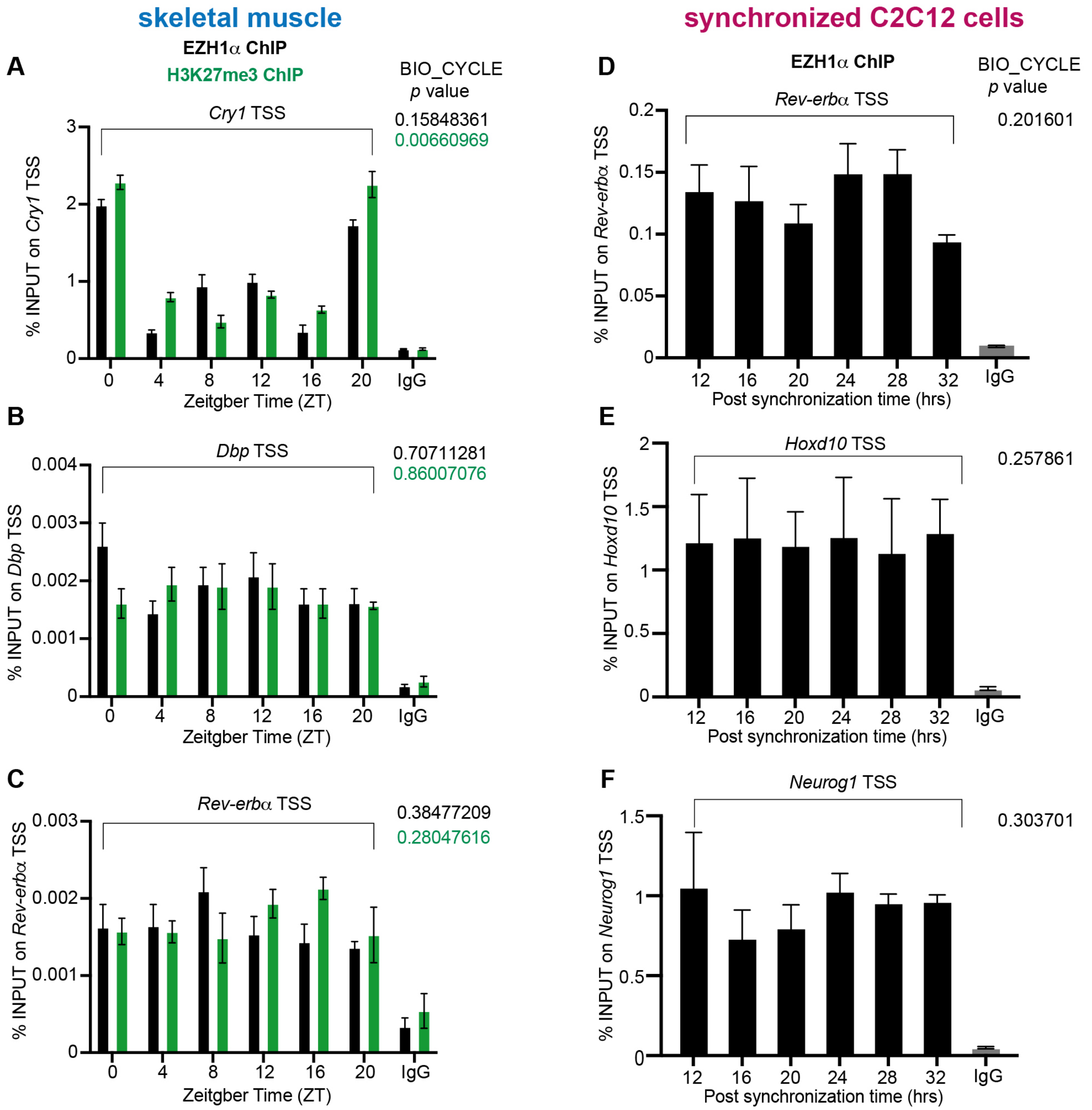

Appendix Figure S3. Oscillational recruitment of Ezh1α and H3K27me3 deposition at transcription start site (TSS) of clock genes in muscle tissue. a-c ChIP experiment of EZH1α and H3K27me3 at transcription start site (TSS) of (**a**) *Cry*1 (**a**), (**b**) *Dbp* (**b**) and (**c**) *Rev-erbα* (**c**) was performed using cross-linked chromatin from gastrocnemius muscle tissue. **d-f** ChIP experiment of EZH1α at transcription start site (TSS) of *Rev-erbα* (**c**), *Hoxd10* (**d**), and *Neurog1* (**e**) was performed using cross-linked chromatin from synchronized C2C12 cells. Immunoprecipitated DNA was quantified by qPCR at the indicated zeitgeber times (ZT, hours after lights on) (n= 3 per time point). Error bars represent ± SEM from three independent experiments. IgG represents ChIP experiment performed with an isotype-matched control immunoglobulin (normal rabbit IgG) to Ezh1α or H3K27me3. Circadian *P* values on the right of each panel represent rhythmic occupancy of EZH1 and H3K27me3 using the non-parametric test Bio-cycle, where $p < 0.05$ is considered statistically significant, incorporating a window of 24hr in muscle tissue and a window of 32hr in C2C12 cells.

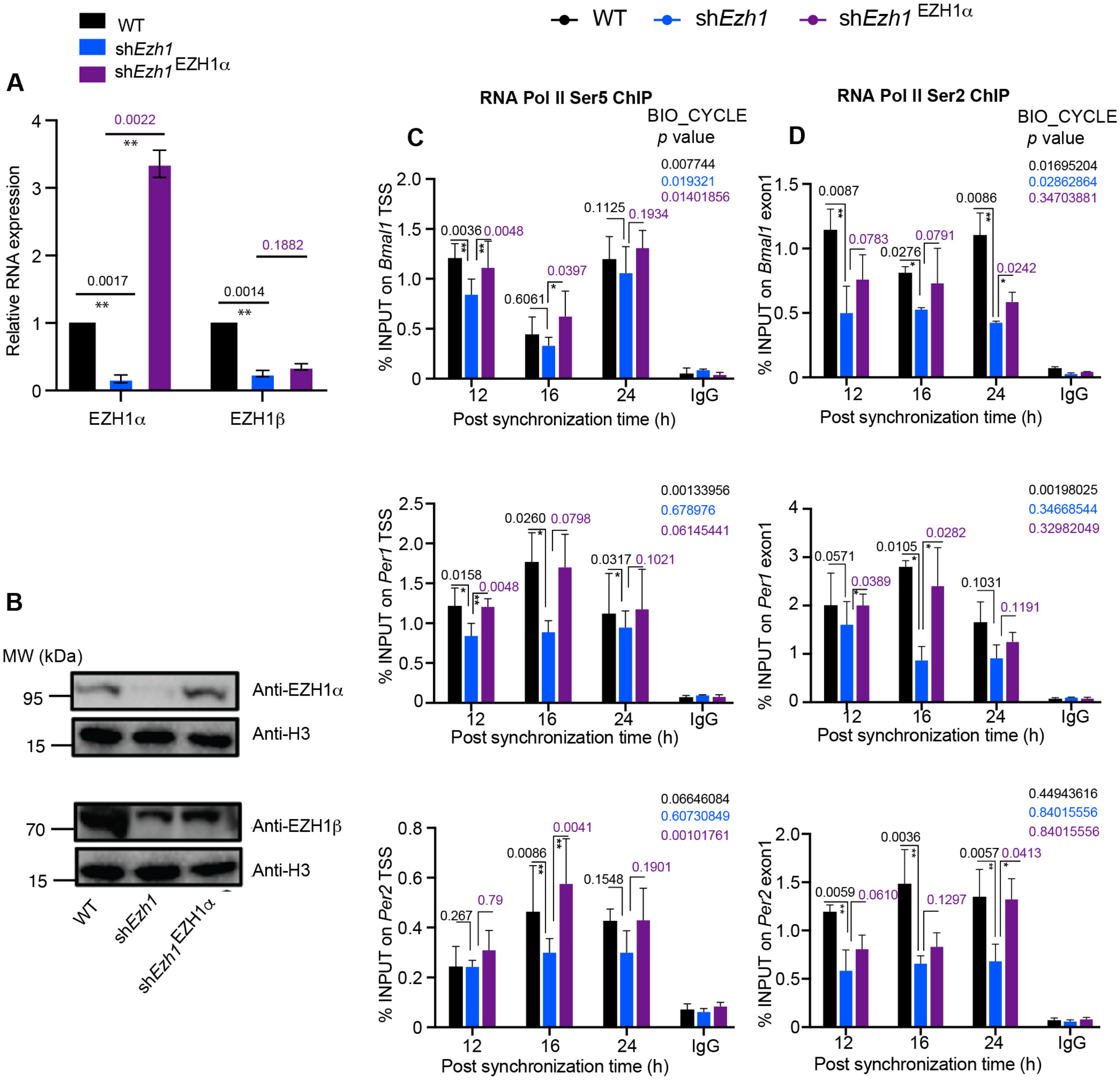

Appendix Figure S4. EZH1 is required for oscillational occupancy of both RNA Pol II initiation and elongation form around genomic loci of core clock genes. (A, B) Transcript (**A**) and protein (**B**) levels of Ezh1α/β were measured in WT, *shEzh1,* and *shEzh1*$^{\mathrm{EZH1\alpha}}$ C2C12 cell lines using RT-qPCR and WB analysis respectively. H3 was used as a loading control for WB analysis. Error bars represent ± SEM from three independent experiments. $**p < 0.01$; by two-tailed t test. (**C-D**) ChIP experiment from Pol ll Ser-5 (**C**) and Pol II Ser-2 (**D**) at transcription start site (TSS) and gene body (Exon1) respectively of *Bmal1* (top panel), *Per1* (middle panel), and *Per2* (right panel) during different circadian time points in synchronized C2C12 from WT, *shEZH1* and *shEZH1*^{Ezh1a} (n= 3 per time point). Immunoprecipitated chromatin was quantified by qPCR and normalized to input. IgG represents ChIP experiment performed with an isotype-matched control immunoglobulin (normal rabbit IgG) to Pol ll Ser-5 and Pol II Ser-2. Error bars represent ± SEM of three independent replicates. $*p < 0.05$; $**p < 0.01$; by two-tailed t test. Circadian P values ($p < 0.05$ or less) represent rhythmic transcripts using the non-parametric test Bio-cycle, incorporating a window of 24hr.

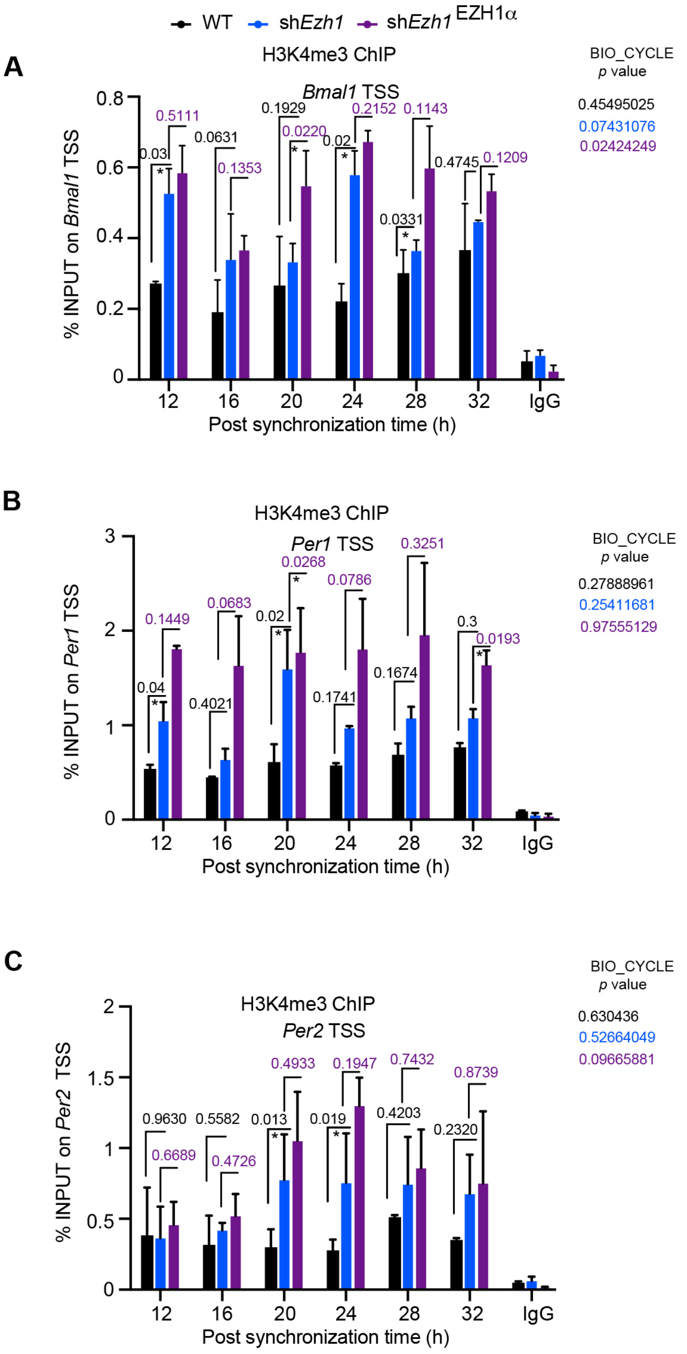

Appendix Figure S5. Enhanced occupancy profile of H3K4me3 surrounding core clock genes following EZH1 depletion. (A-C) ChIP experiment of H3K4me3 at transcription start site (TSS) respectively of *Bmal1* (**A**), *Per1* (**B**), and *Per2* (**C**) during different circadian time points in synchronized C2C12 from WT, *shEZH1* and $shEZH1^{Ezh1a}$ (n= 3 per time point). Immunoprecipitated DNA was quantified by qPCR and normalized to input. IgG represents ChIP experiment performed with an isotype-matched control immunoglobulin (normal rabbit IgG) to H3K4me3. Error bars represent ±SEM of three independent replicates. **p* < 0.05; by two-tailed *t* test. Circadian *P* values (*p* < 0.05 or less) represent rhythmic transcripts using the non-parametric test Bio-cycle, incorporating a window of 32hr.

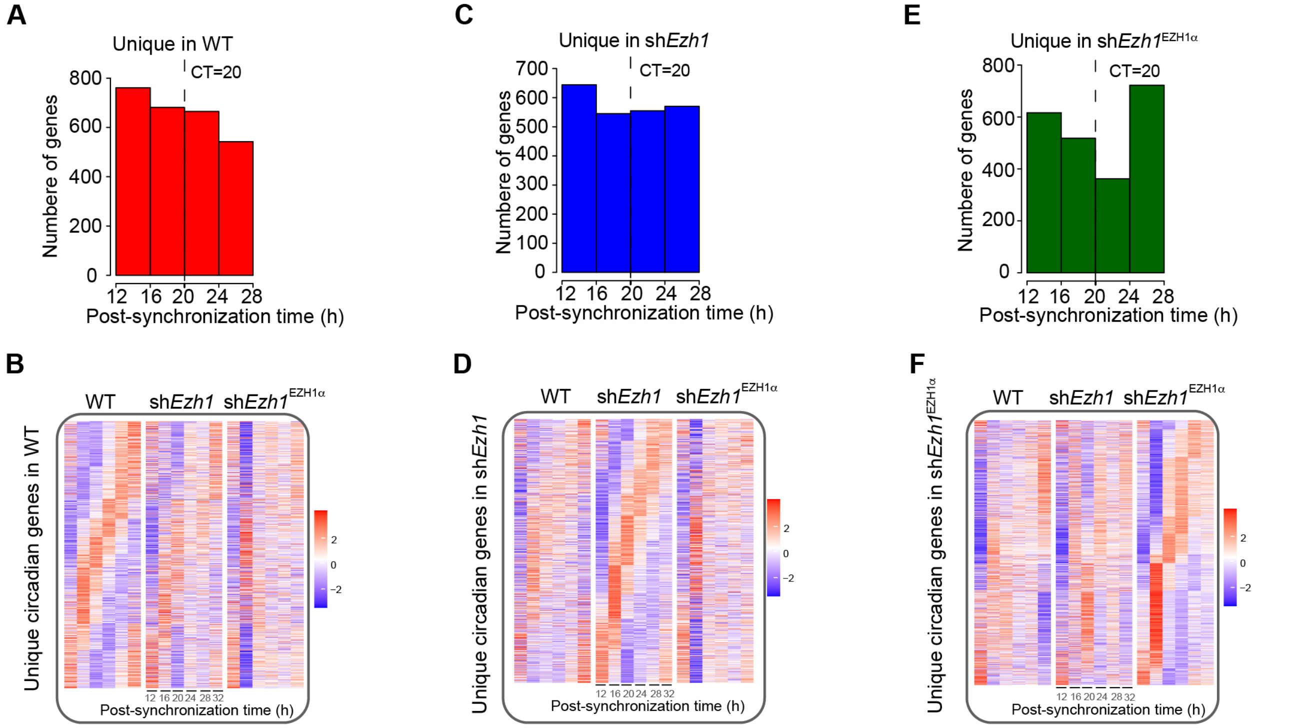

Appendix Figure S6. Peak phase distribution and heatmap analysis of unique circadian genes in wild type (WT), sh*Ezh1* and sh*Ezh1*$^{\mathbf{EZH1\alpha}}$ conditions. (A, C and **E)** Peak phase distribution of unique circadian genes in WT (**A**), sh*Ezh1* (**C**) and sh*Ezh1*$^{EZH1\alpha}$ (**E**). (**B, D** and **F)** Heatmap profile of unique circadian transcripts in WT (**B**), sh*Ezh1* (**D**) and sh*Ezh1*$^{EZH1\alpha}$ (**F**) conditions.

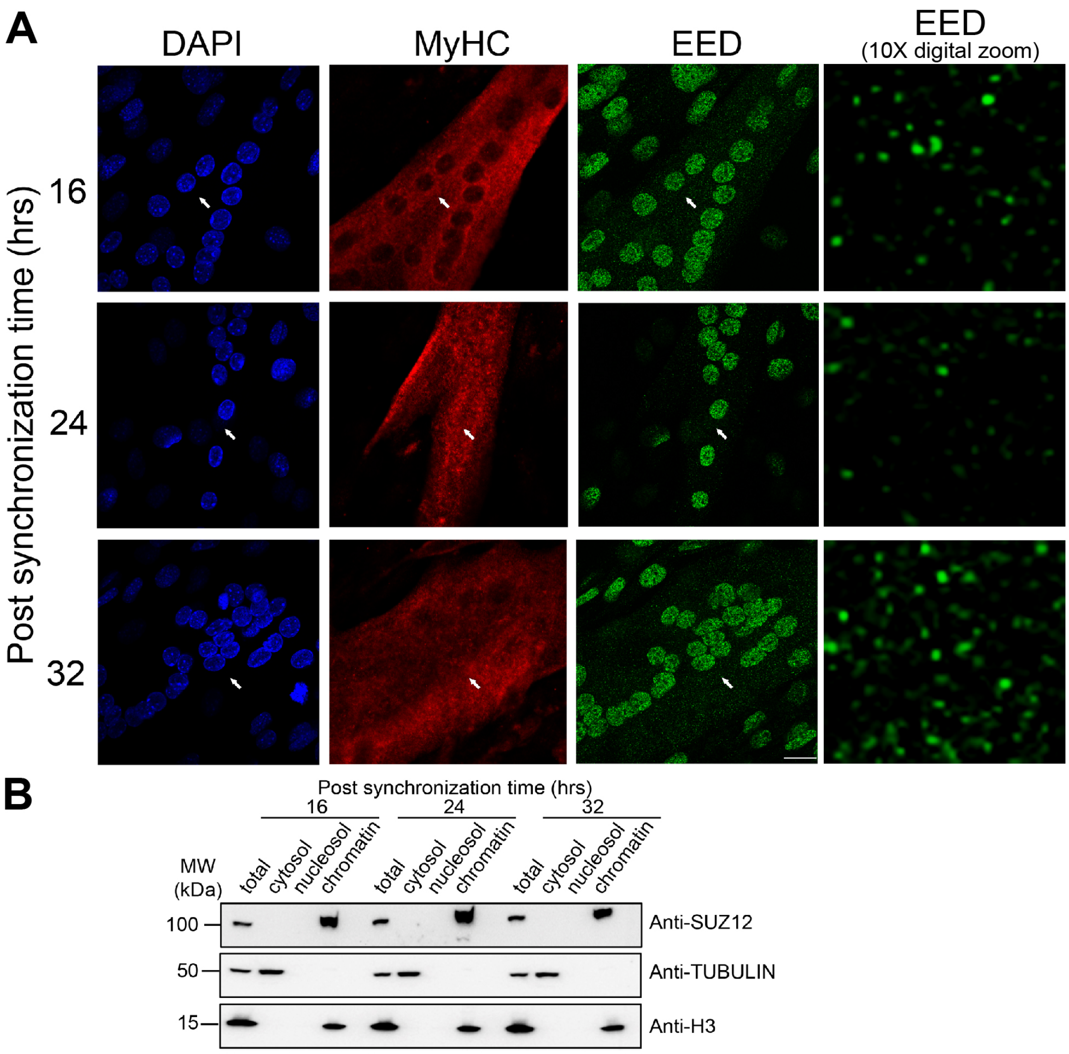

Appendix Figure S7. SUZ12 constantly bounds to chromatin alongside the circadian cycle. (A) Representative immunofluorescence of EED (green) and MyHC (red) at indicated time points in C2C12 cells, DNA was counterstained with DAPI (blue). Scale bar =100 μm. White arrows indicate zoomed reign of interest (10X digital zoom), present in the rightmost panel. **(B)** Immunoblot analysis of SUZ12 distribution in the cytosol, nucleosol, and chromatin-bound fractions at indicated circadian time points in wild-type C2C12 cells. Tubulin or H3 was used as cytosol and chromatin fraction control, respectively.

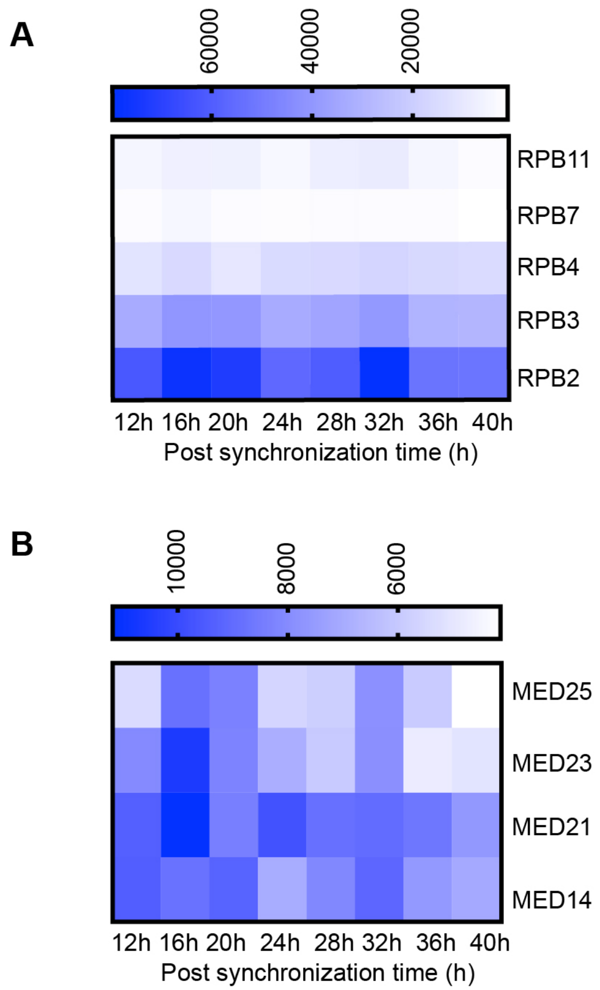

Appendix Figure S8. Dynamic interaction between EZH1α and RNA Pol II associated components. (A, B) Immunoprecipitation coupled with quantitative mass spectrometry analysis indicates differential interaction between EZH1α and subunits of RNA Pol II (**A**) and Mediator proteins (**B**) at different time points.

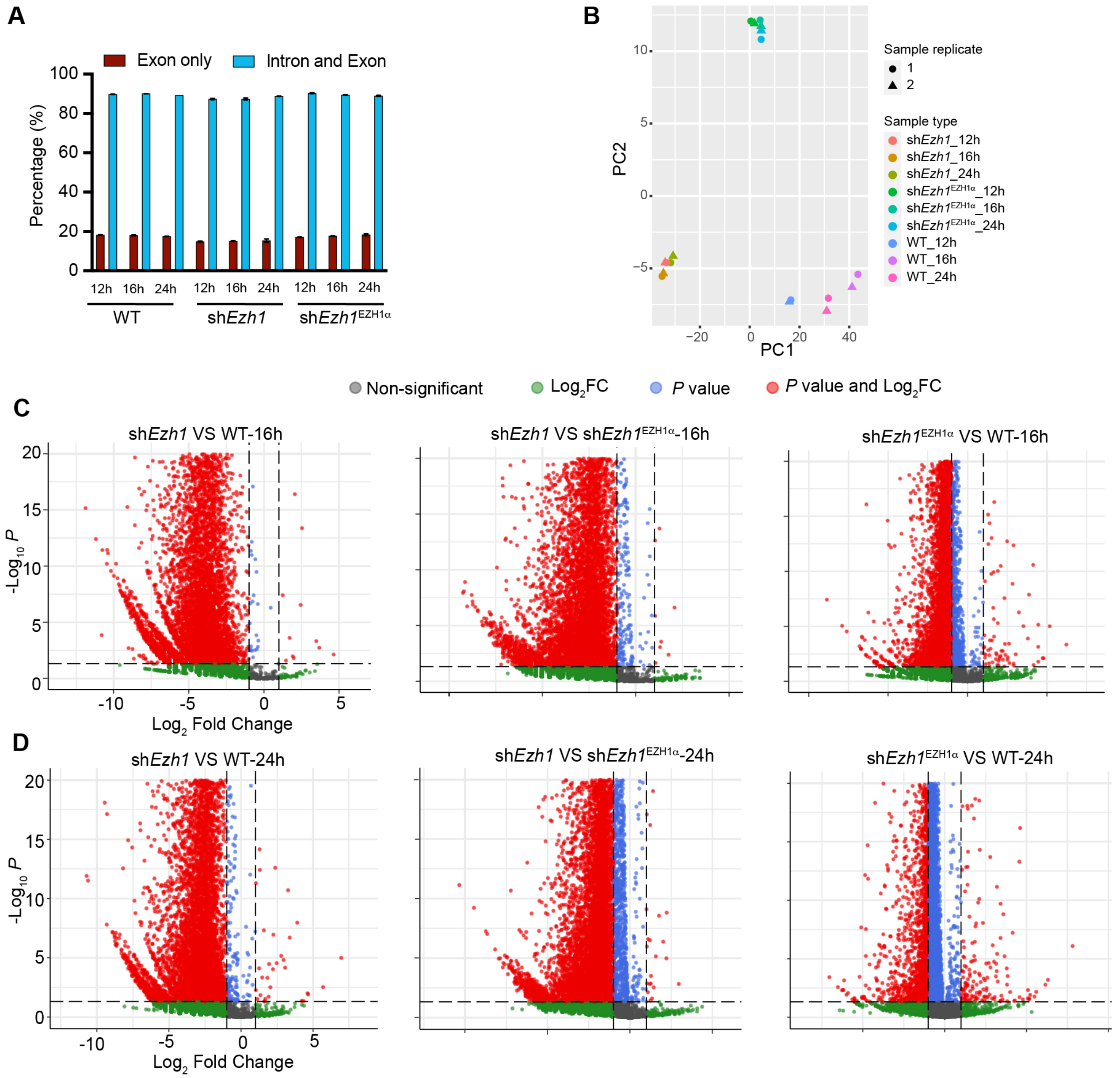

Appendix Figure S9. Quality control of TT-seq and differentially expressed nascent transcripts profile in comparison with indicated biological conditions. (A) Percentage of reads mapping to exonic or intronic regions from TT-seq in WT, sh*Ezh1* and sh*Ezh1*^{EZH1a} under indicated time points. **(B)** Principal component analysis (PCA) plot of the TT-seq data from all samples used in this study. **(C, D)** Volcano plot representation of differential expression profile of nascent transcripts in comparison with different biological conditions at indicated post-synchronization time points. The statistical analysis in panel (**C**) and (**D**) were performed using DESeq2 as described in Method section.

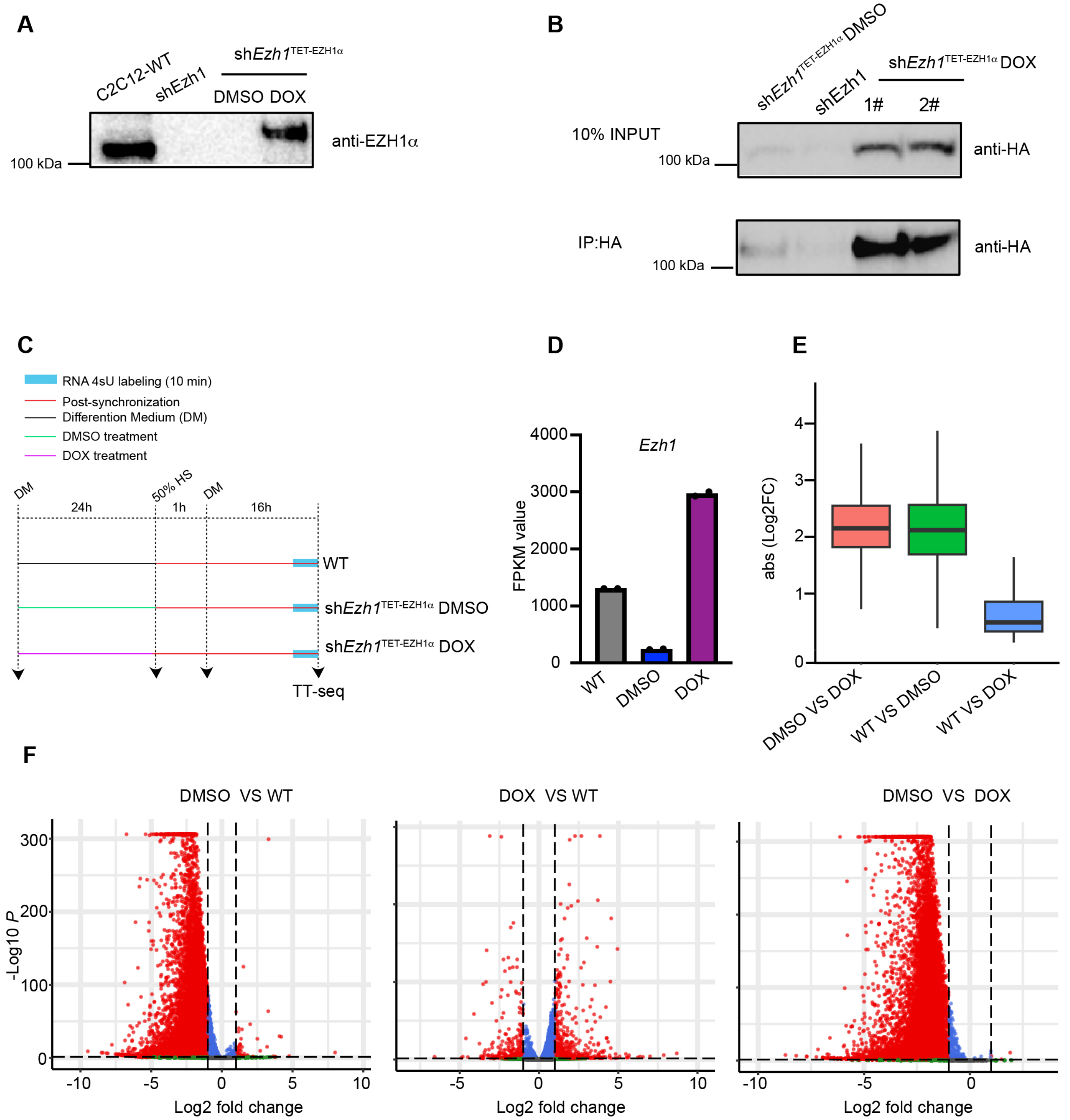

Appendix Figure S10. Restoration of expression levels to normal by transient EZH1 expression in sh*Ezh1* knock-down background. (A) The protein levels of Ezh1α were assessed through Western blot (WB) analysis in various C2C12 cell lines, including WT, *shEzh1 and shEzh1*$^{\text{TET-EZH1α}}$ (treated with DMSO or DOX) transgenic cells. In the WB analysis, the band observed in the WT lane corresponds to the endogenous EZH1α protein. In contrast, the band in the DOX lane represents the HA-EZH1α. **(B)** Different cell lines were utilized to immunoprecipitate the HA-EZH1α protein using HA conjugated agarose beads. Subsequently, the HA tagged proteins were visualized using Anti-HA. **(C)** Experimental design illustrating TT-seq samples collection at indicated conditions. **(D)** FPKM value of EZH1 in WT, *shEzh1*$^{\text{TET-EZH1α}}$ treated with DMSO (DMSO) and *shEzh1*$^{\text{TET-EZH1α}}$ treated with DOX (DOX). **(E)** Absolute fold changes of significantly differential genes in comparison with indicated biological conditions at 16h. 14117, 13907 and 4364 genes were analyzed in comparison with indicated biological conditions at 16h. Detailed minima, maxima, centre, bounds of box and whiskers, and percentile

value have been provided in Dataset EV8. **(F)** Volcano plot representation of differential expression profile of nascent transcripts in comparison with different biological conditions at 16h. The statistical analysis in panel (**F**) was performed using DESeq2 as described in Method section.

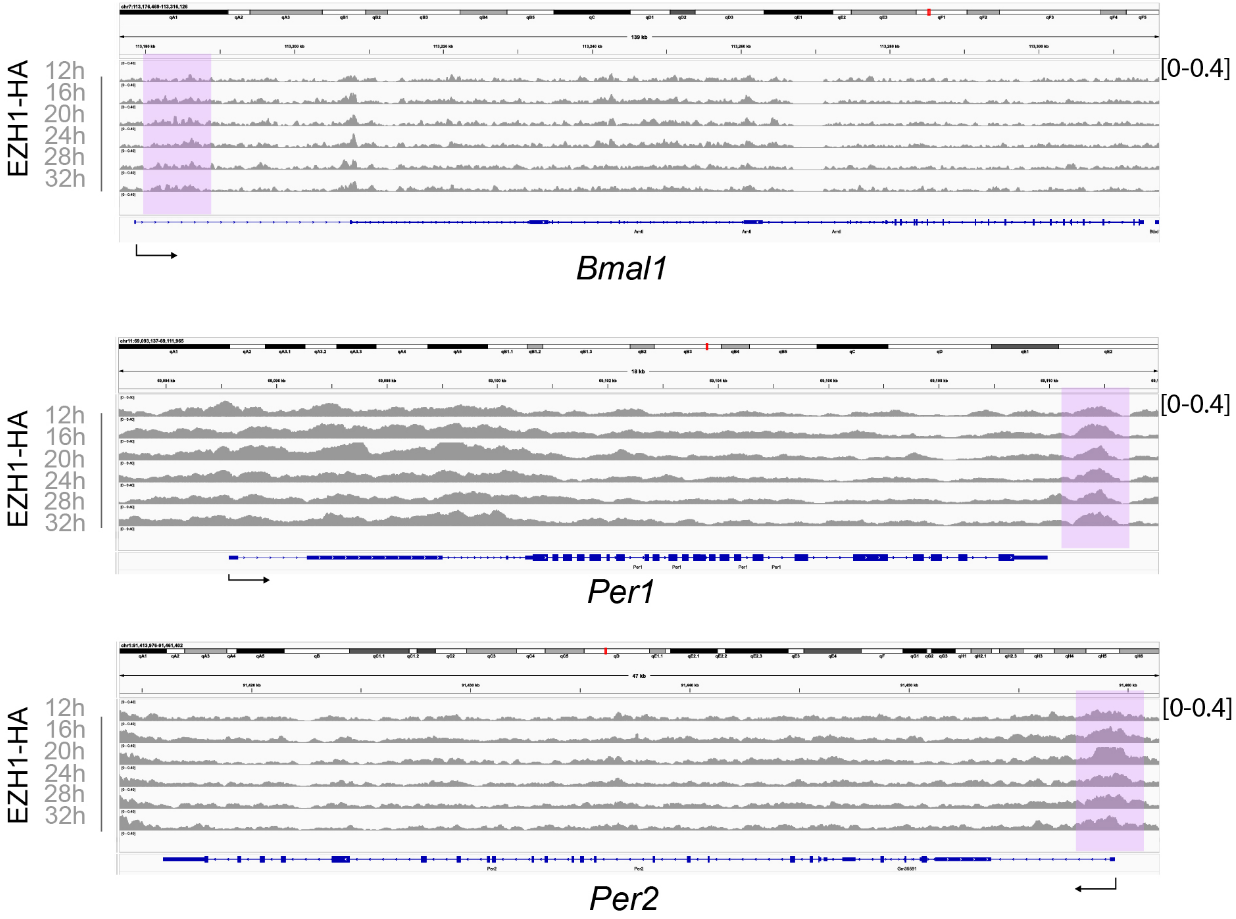

Appendix Figure S11. Direct association of EZH1α and H3K27me3 around *Bmal1*, *Per1* and *Per2* loci. IGV track of EZH1 peak distribution around *Bmal1, Per1 and Per2* genes loci. Region around Transcription Start Site (TSS) was highlighted.

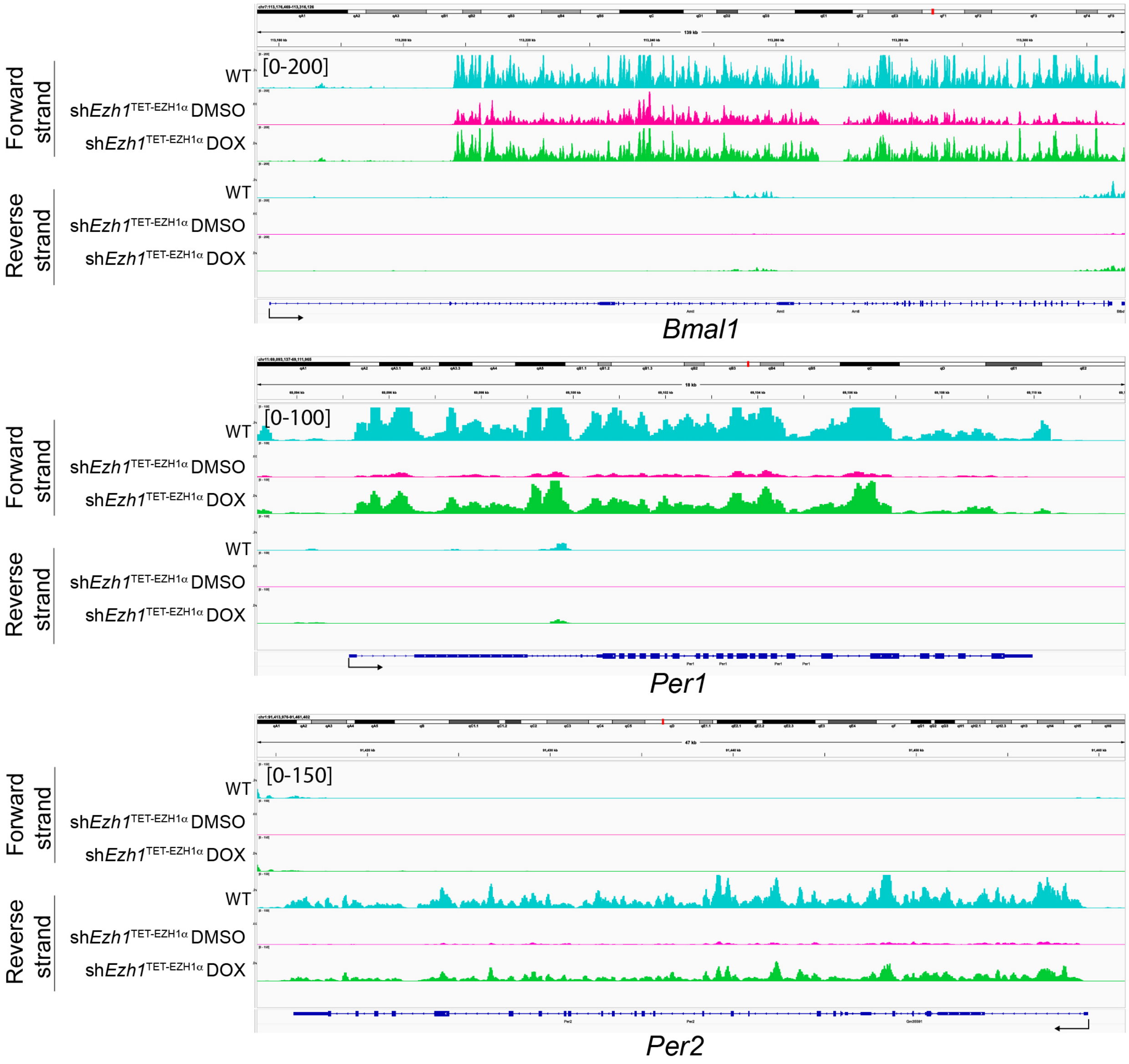

Appendix Figure S12. EZH1α is required for *Bmal1*, *Per1* and *Per2* expression. IGV track of TT-seq signal around *Bmal1, Per1 and Per2* genes loci under indicated genetic background.

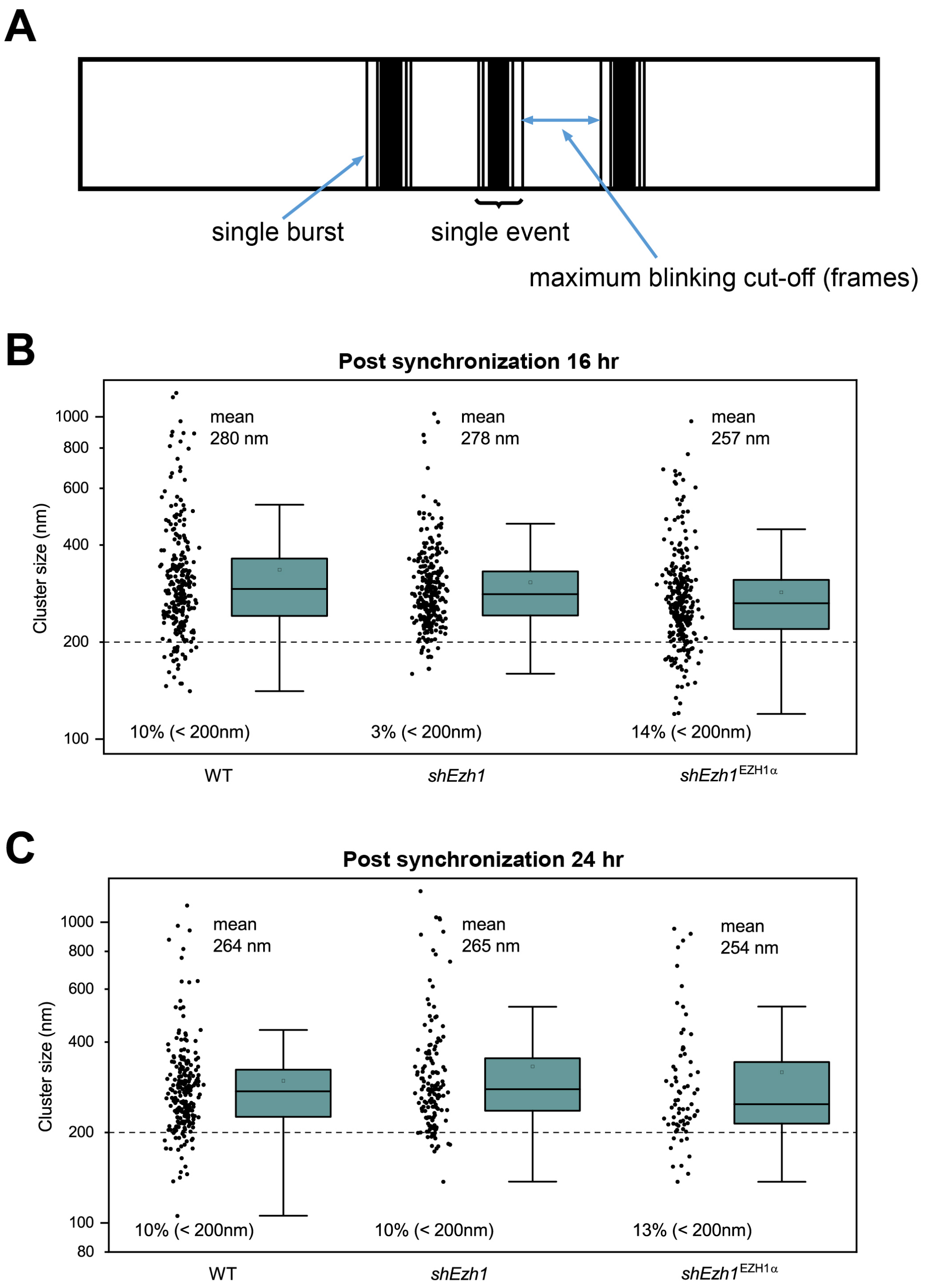

Appendix Figure S13. Quantification of the cluster size of RNA Pol II in WT, *shEzh1* and *shEzh1*$^{EZH1\alpha}$ at indicated time points. (A) Schematic illustration of measured parameters: single burst (fluorescence signal in one frame), single event of RNA Pol II-Dendra2 clustering and maximum blinking cut-off. **(B, C)** Dot plots superimposed box plots of the cluster size in WT, *shEzh1* and *shEzh1*$^{EZH1\alpha}$. The mean cluster sizes and the percentages of the small (<200 nm) clusters are indicated in the top and bottom of each panel. 258, 293 and 283 clusters were analyzed for in WT, *shEzh1* and *shEzh1*$^{EZH1\alpha}$ respectively in panel (**B**). 228, 146 and 69 clusters were analyzed for WT, *shEzh1* and *shEzh1*$^{EZH1\alpha}$ respectively in panel (**C**). Detailed minima, maxima, centre, bounds of box and whiskers, and percentile value for panel (**B**) and (**C**) have been provided in Dataset EV8.

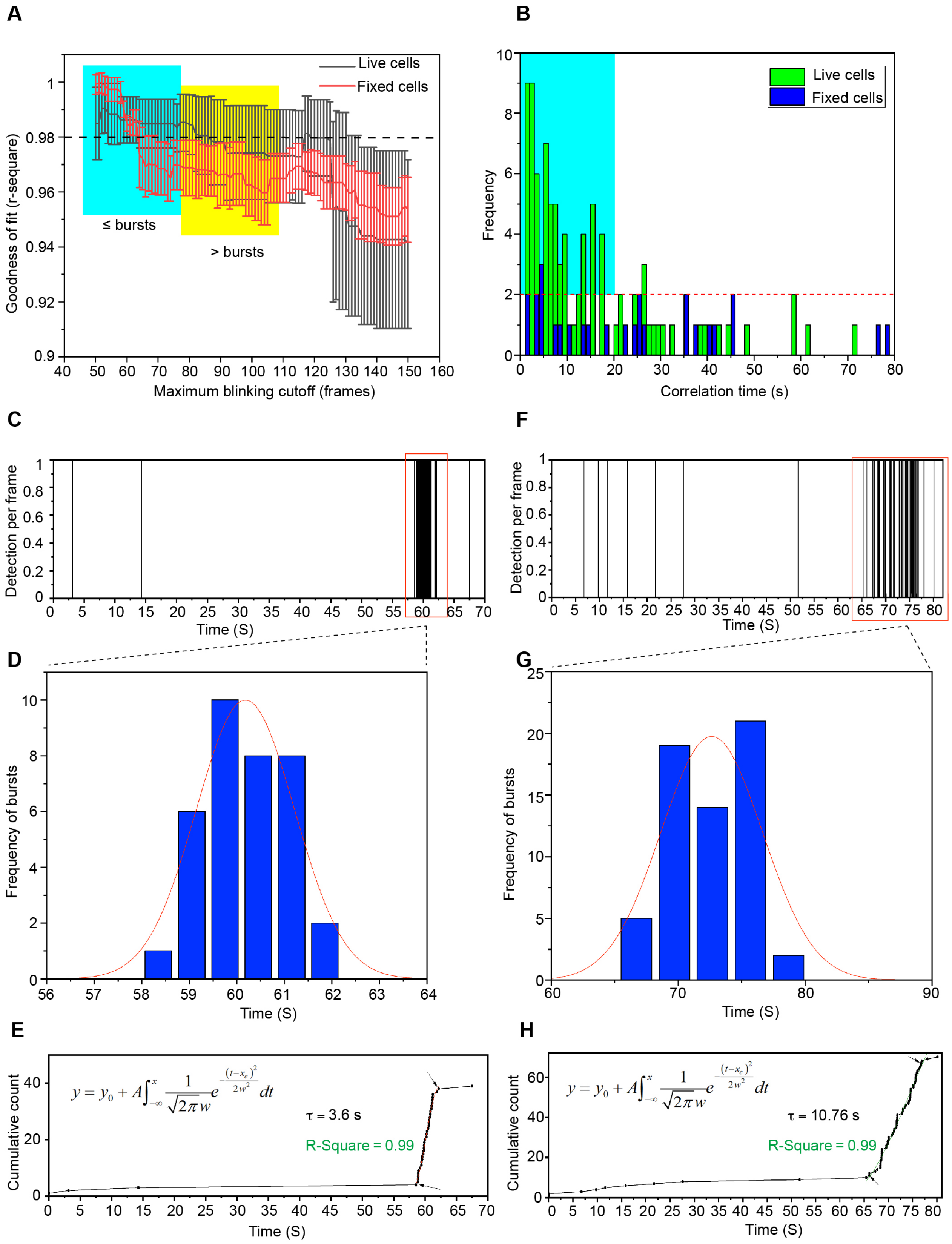

Appendix Figure S14. Distinction between physiological clustering of RNA Pol II and photophysical dynamics of Dendra2. (A) Average count per burst was normalized to compare goodness of fit (R-square value) as a function of maximum blinking cutoff (frames) in live and

fixed cells. Yellow square indicates events selected for lifetime analyses. The number of bursts detected by using a maximum blinking cut-off of 75 frames and below were less than 10 bursts. This number of bursts was not enough for a reliable fitting of the data to the cumulative distribution function. Thus, we choose 100 frames as a maximum blinking cut-off for our analysis. **(B)** Frequency distribution of the correlation time of the burst events in live and fixed cells. Cyan square regions define events for further analyses. As shown in (**A**), the events due to single molecule photo physics in fixed cells showed R-squared values below the set threshold of 0.98. We normalized the data based on the number of bursts where we considered 2500 bursts in in live and in fixed cells to calculate the correlation time. As shown in (**B**), our analysis (maximum blinking cutoff = 100 frames and R-squared value ≥ 0.98) showed that the correlation time obtained from live cells can be distinguished from fixed cells by considering correlation time values below 20 seconds. To further remove any contribution of the photophysical phenomenon, we removed the correlation time values if their frequencies is smaller than 20% of the maximum frequency. **(C)** and **(F)**Representative time traces for two selected small RNA Pol II clusters. **(D)** and **(G)** Frequency distributions of bursts shown in (**C**) and (**F**), respectively. The red lines show the fitting to Gaussian (normal) distribution. **(E)** and **(H)** Cumulative count of bursts shown in (**C**) and (**F**), respectively. The black dot lines indicate the fitting of the cumulative count to normal cumulative distribution function, as calculated by the equation in the left side of each panel.

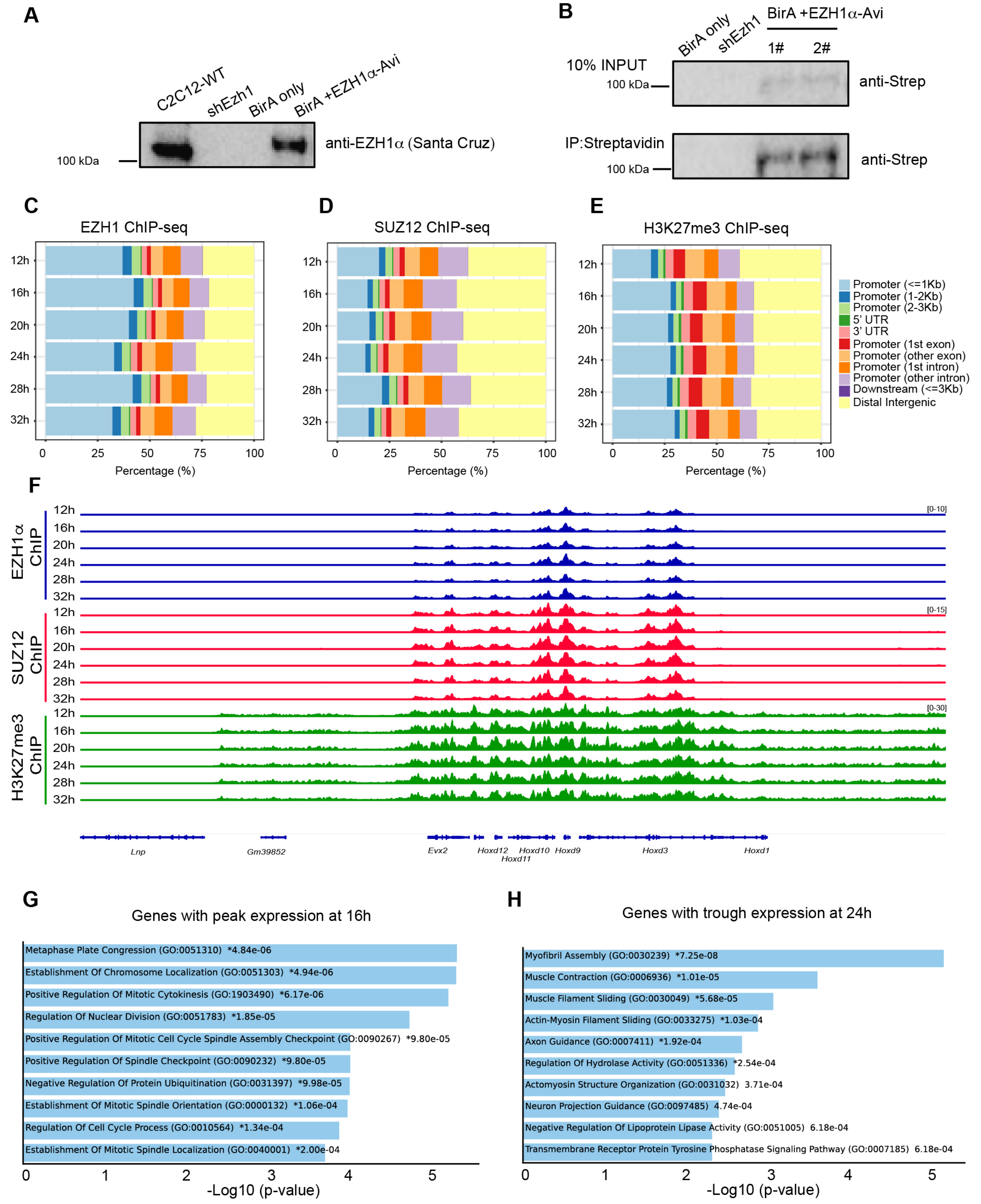

Appendix Figure S15. Genomic features of EZH1α, SUZ12, and H3K27me3 ChIP-seq. (A) Protein levels of Ezh1α were assessed through Western blot (WB) analysis in various C2C12 cell lines, including WT, *shEzh1, shEzh1*^{BirA}, and *shEzh1*$^{BirA+EZH1\alpha-Avi}$ transgenic cells. In the WB analysis, the band observed in the WT lane corresponds to the endogenous EZH1α protein. In contrast, the band in the BirA+EZH1α-Avi Lane represents the biotinylated form of EZH1α. **(B)** Different indicated cell lines were utilized to immunoprecipitate the biotinylated EZH1α protein

using streptavidin resin. Subsequently, the biotin-labeled proteins were visualized using Streptavidin-HRP. **(C-E)** Genomic features were annotated for the ChIP-seq peaks of EZH1α(**C**), SUZ12(**D**), and H3K27me3(**E**). **(F)** IGV track of EZH1α, SUZ12, and H3K27me3 peak distribution around *Hoxd* genes cluster loci. **(G, H)** Gene ontology (GO) enrichment analysis of genes that expressed with the highest level (peak) or lowest level (trough) at 16h (**G**) and 24h (**H**). Statistical analysis was performed using EnrichR.

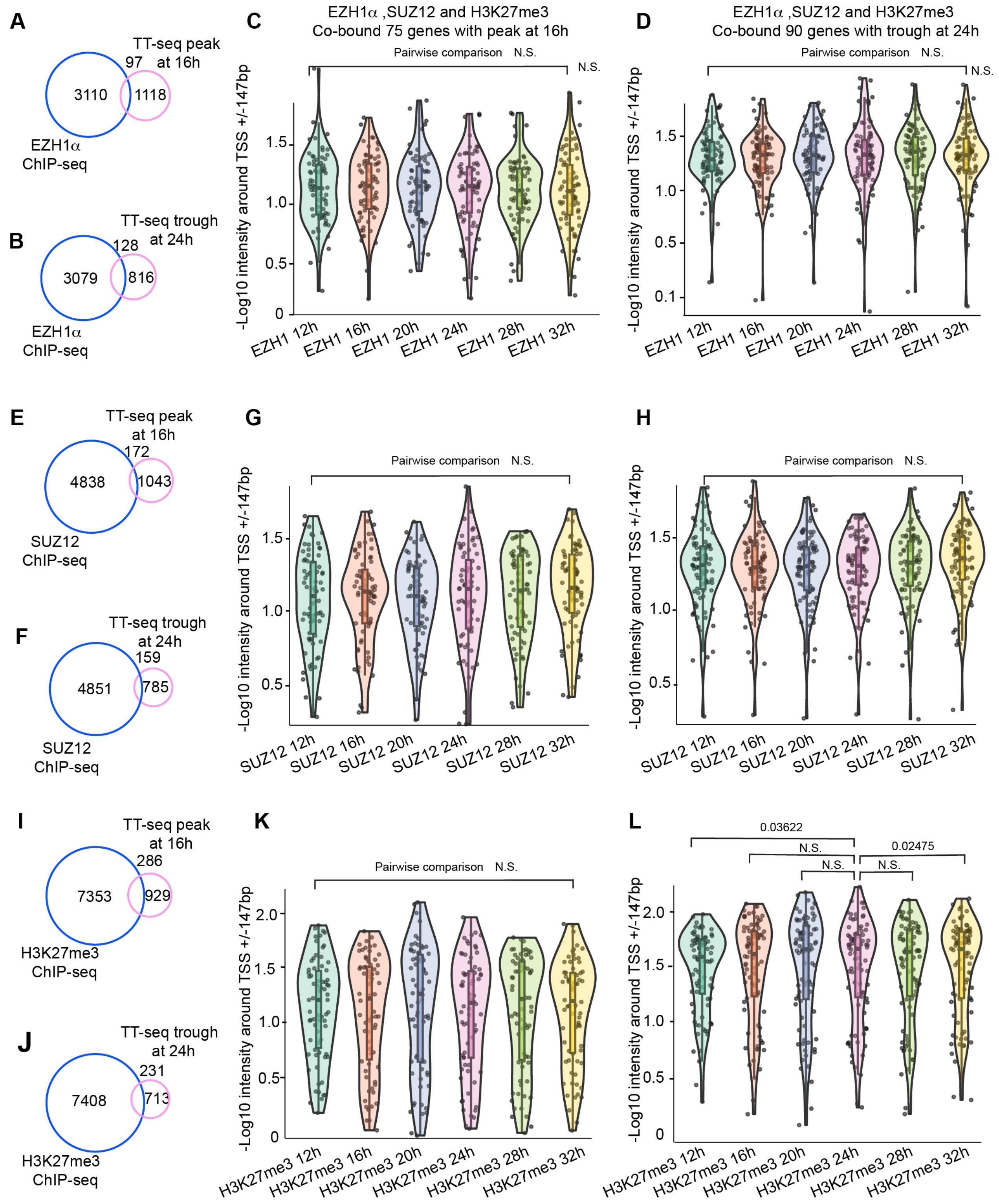

Appendix Figure S16. Distinct occupancy profile of EZH1α, SUZ12 and H3K27me3 around the 1st nucleosome of activated and repressive targets. (A, B) Venn diagram of overlap between EZH1 bound targets and genes expressed with the highest level (peak) or lowest level (trough) at 16h (**A**) and 24h (**B**), as determined by TT-seq in wild-type (WT) synchronized C2C12 cells. **(C, D)** Violin plots illustrate the peak intensity of EZH1 ChIP-seq peaks surrounding the transcription start site of genes expressed with the highest level (peak) or lowest level (trough) at 16h (**C**) and

24h (**D**) co-bound by EZH1α, SUZ12 and H3K27me3 encompassing upstream and downstream regions spanning one nucleosome. Each dot on the plot represents the average peak intensity around each gene. Detailed minima, maxima, centre, bounds of box and whiskers, and percentile value have been provided in Dataset EV8. **(E, F)** Venn diagram of overlap between SUZ12 bound targets and genes expressed with the highest level (peak) or lowest level (trough) at 16h (**E**) and 24h (**F**), as determined by TT-seq in wild-type (WT) synchronized C2C12 cells. **(G, H)** Violin plots illustrate the peak intensity of SUZ12 ChIP-seq peaks surrounding the transcription start site of genes expressed with the highest level (peak) or lowest level (trough) at 16h (**G**) and 24h (**H**) bound by EZH1α, SUZ12 and H3K27me3 encompassing upstream and downstream regions spanning one nucleosome. Each dot on the plot represents the average peak intensity around each gene. Detailed minima, maxima, centre, bounds of box and whiskers, and percentile value have been provided in Dataset EV8. **(I, J)** Venn diagram of overlap between H3K27me3 bound targets and genes expressed with the highest level (peak) or lowest level (trough) at 16h (**I**) and 24h (**J**), as determined by TT-seq in wild-type (WT) synchronized C2C12 cells. **(K, L)** Violin plots illustrate the peak intensity of EZH1 ChIP-seq peaks surrounding the transcription start site of genes expressed with the highest level (peak) or lowest level (trough) at 16h (**K**) and 24h (**L**) occupied by EZH1α, SUZ12, and H3K27me3 encompassing upstream and downstream regions spanning one nucleosome. Each dot on the plot represents the average peak intensity around each gene. Detailed minima, maxima, centre, bounds of box and whiskers, and percentile value have been provided in Dataset EV8. To identify significant differences in peak intensity across time points, pairwise Wilcoxon rank-sum tests were conducted. The *P* values from these tests were adjusted for multiple comparisons using the Benjamini-Hochberg method.
