# Supplementary figures and images for: PRC2-EZH1 contributes to circadian gene expression by orchestrating chromatin states and RNA polymerase II complex stability
Source: EMBO J. 2024 Oct 21;43(23):6052–75. doi: 10.1038/s44318-024-00267-2 (PMC11612306; doi:10.1038/s44318-024-00267-2)

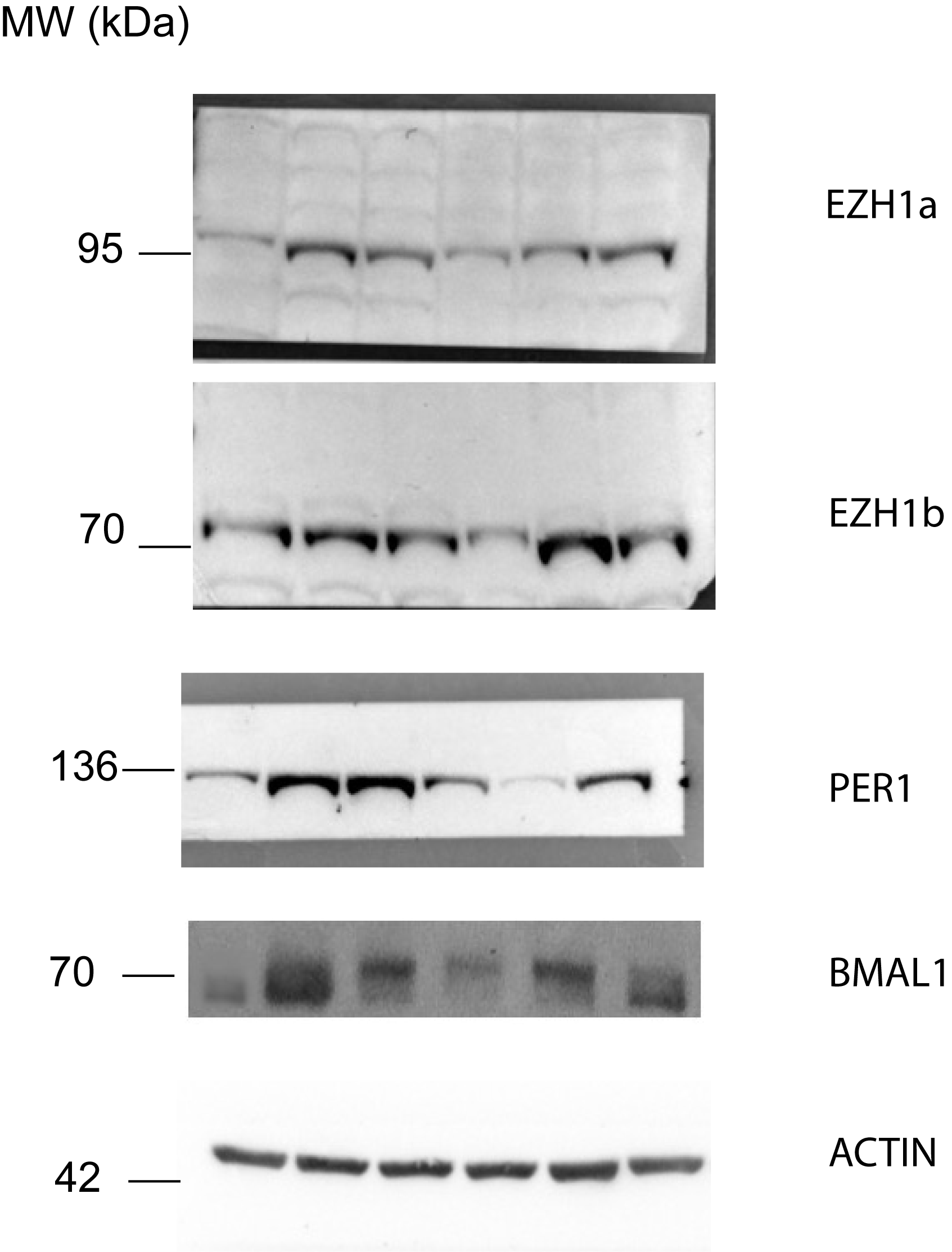

Supplement: Supplementary file 11 — Source data Fig. 1 [file 44318_2024_267_MOESM11_ESM.zip › Figure 1/1B/Figure 1B WB source data.tif]

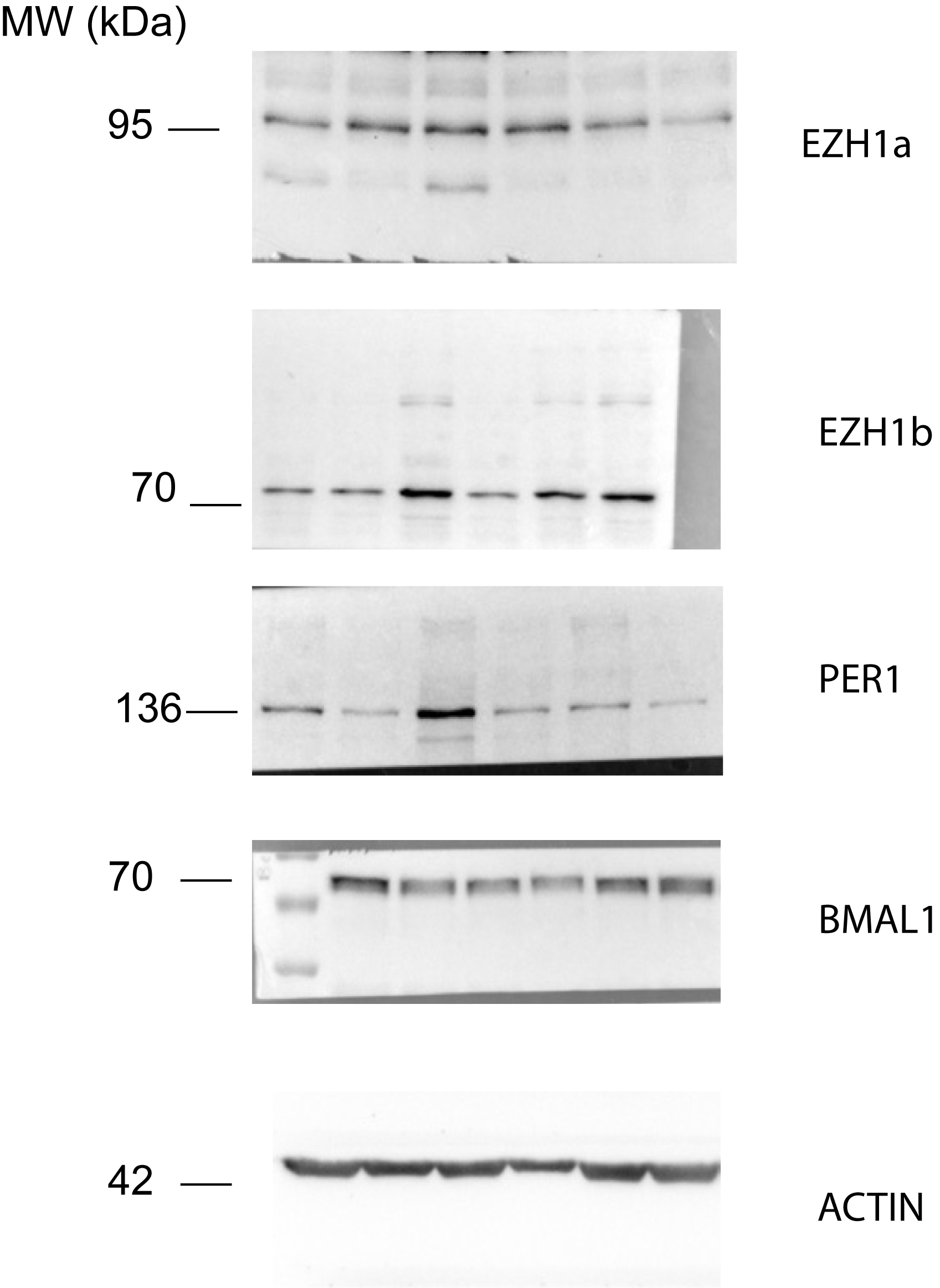

Supplement: Supplementary file 11 — Source data Fig. 1 [file 44318_2024_267_MOESM11_ESM.zip › Figure 1/1E/Figure 1E WB source data.tif]

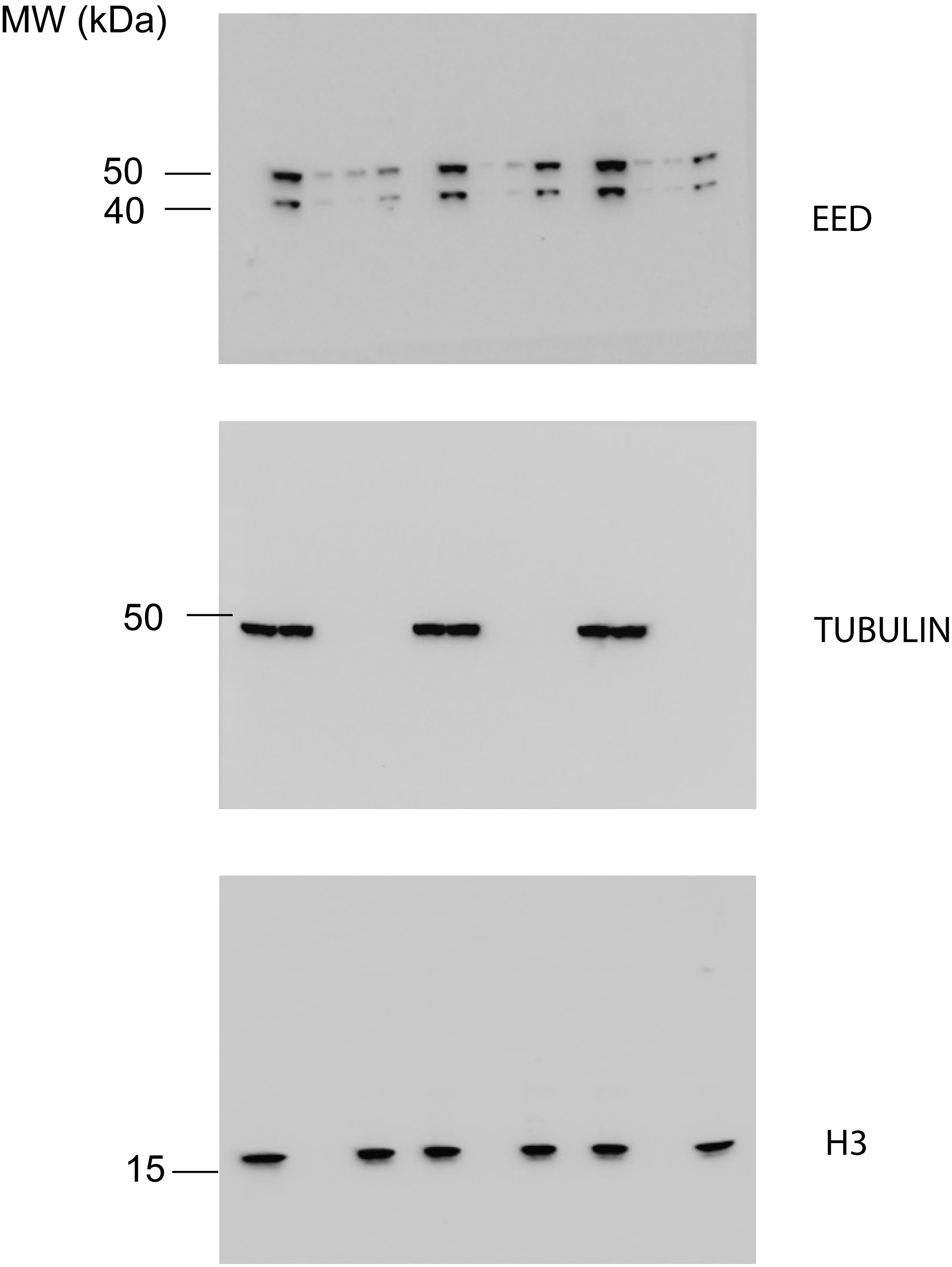

Supplement: Supplementary file 14 — Source data Fig. 4 [file 44318_2024_267_MOESM14_ESM.zip › Figure 4/Fig4D/Figure 4D source data.tif]
